# Heterophile carbohydrate antigen *N*‐glycolylneuraminic acid as a potential biomarker in patients with hepatocellular carcinoma

**DOI:** 10.1002/cnr2.1831

**Published:** 2023-06-02

**Authors:** Shuji Akimoto, Hiroyuki Tahara, Senichiro Yanagawa, Kentaro Ide, Yuka Tanaka, Tsuyoshi Kobayashi, Hideki Ohdan

**Affiliations:** ^1^ Department of Gastroenterological and Transplant Surgery Graduate School of Biomedical & Health Sciences, Hiroshima University Hiroshima Japan

**Keywords:** cytidine‐5′‐monophospho‐NeuAc hydroxylase, hepatocellular carcinoma, *N*‐glycolylneuraminic acid, prognosis, recurrence

## Abstract

**Background and Objectives:**

Hepatocellular carcinoma (HCC) has a high recurrence rate even after radical hepatectomy. More optimal biomarkers may help improve recurrence and prognosis.

**Methods:**

We investigated whether the oncological properties of *N*‐glycolylneuraminic acid (NeuGc) can participate in the prognosis of HCC. We evaluated the NeuGc antigen (Ag) expression in the HCC tissues and measured the preoperative anti‐NeuGc IgG antibodies (Abs) in the sera of the patients with HCC. We compared the clinical characteristics and survival rate in the hepatectomized patients (initial; *n* = 66, recurrent; *n* = 34) with and without the NeuGc Ag or Abs.

**Results:**

Multivariate analyses showed positive expression of NeuGc Ag in HCC tissues (Odds ratio; initial = 6.3, recurrent = 14.0) and higher titers of preoperative anti‐NeuGc Ab (Odds ratio; initial = 4.9; recurrent = 3.8), which could be the predictive factors related to early recurrence. Both the NeuGc Ag‐positive and Ab‐positive groups in the initial hepatectomized patients exhibited significantly shorter recurrent free survival compared to those in the negative groups.

**Conclusions:**

Our findings suggested that anti‐NeuGc Ab titers and NeuGc Ag expression in the HCC tissues can be used as the predictive factors for the postoperative recurrence and prognosis of HCC.

## 1 INTRODUCTION

Sialic acid is a 9‐carbon carbohydrate found in the terminal sugar chains of glycolipids and glycoproteins.^1^ Sialic acid‐containing sugar chains play an important role in cell‐to‐cell communication and cell‐to‐pathogen recognition.^1^ In mammals, the most predominant types of sialic acid are *N*‐acetylneuraminic acid (NeuAc) and *N*‐glycolylneuraminic acid (NeuGc). NeuGc is widely expressed in most mammals but not in normal human tissues.^1^ The NeuGc deficiency in humans is caused by exon deletion in the gene coding for cytidine‐5′‐monophospho (*CMP*)‐NeuAc hydroxylase (*CMAH*), which converts *CMP*‐NeuAc into *CMP*‐NeuGc, resulting in a markedly truncated protein lacking amino acid residues necessary for enzyme activity. The human *CMAH* gene, also known as *CMAH* pseudogene (*CMAHP*), was pseudogenized and inactivated by the deletion event.^2^ This mutation is homozygous among human populations but is absent in great apes.^3^ Therefore, the natural anti‐NeuGc immunoglobulin (Ig) G antibodies (Abs) were reportedly detectable in 85% of healthy humans.^4^ Furthermore, the sugar chains expressed on the cell surface do not only qualitatively and quantitatively change during tumor development but also play an important role in cancer cell infiltration and metastasis.

Recently, NeuGc antigen (Ag) was discovered to be expressed in lung, breast, colon cancer, melanoma, retinoblastoma, and hepatocellular carcinoma (HCC) patients.^5^, ^6^, ^7^, ^8^ One study found that non‐small cell lung cancer patients with NeuGc Ag expression have a significantly low progression‐free survival rate.^5^ There is a similar paper reporting the possibility of NeuGc containing glycans as a diagnostic biomarker for patients with ovarian cancer.^9^ However, no studies have analyzed the oncological effects of NeuGc Ag expression and anti‐NeuGc Abs to predict HCC prognosis. NeuGc has been reported to be useful as a prognostic and diagnostic biomarker in other carcinomas, and may also be useful in hepatocellular carcinoma. Thus, we aimed to determine whether the NeuGc Ag expression in hepatectomized tissues and anti‐NeuGc Ab titers in the serum may affect HCC prognosis separately for initial and recurrent hepatectomized patients. Our findings may provide new insights to the current research on predicting the recurrence and prognosis in HCC patients. Prediction of the risk of recurrence of HCC may lead to improvement of prognosis of HCC if adjuvant treatment such as molecular target therapy can be given to the high‐risk group for recurrence.

## 2 MATERIALS AND METHODS

### 2.1 Study patients

Between April 2014 and December 2015, 100 patients (of these 100 cases, the initial (*n* = 66) and recurrent (*n* = 34) cases were not overlapped) were hepatectomized for HCC at Hiroshima University Hospital. As a control group, 50 healthy living liver‐transplant donors were preoperatively diagnosed as tumor‐free by computed tomography. This study was performed following the Declaration of Helsinki and approved by the Institutional Review Board of Hiroshima University (approval number: E 1580). Informed consent was obtained from all participants.

### 2.2 Blood and liver tissue samples

Blood samples were preoperatively collected from HCC patients and healthy living liver transplant donors. The resected liver tissues were macroscopically divided into normal and tumor tissues, and then were cryopreserved.

### 2.3 Immunohistochemistry assay

Chicken anti‐NeuGc monoclonal IgG Abs (HU/Ch2‐7; Pharma Foods International Co., Ltd., Kyoto, Japan) were used for the detection of NeuGc Ag in the hepatectomized liver tissues.^10^, ^11^ HU/Ch2‐7, which reacted strongly with NeuGc Ag, was specifically directed toward an antigenic epitope containing NeuGc.^11^

The sections were incubated with HU/Ch2‐7 in phosphate‐buffered saline (PBS) overnight. Biotin‐conjugated goat anti‐chicken IgY (H&L) polyclonal Ab (Abcam, Cambridge, USA) in PBS was added and incubated for 30 min. The sections were incubated with horseradish peroxidase‐conjugated streptavidin (Histofine SAB‐PO Kit; Nichirei Biosciences, Tokyo, Japan) for 30 min. Peroxidase activity was analyzed by staining the sections with 3,3′‐diaminobenzidine (Muto Pure Chemicals, Tokyo, Japan) for 10 min.^12^ The sections were examined and photographed at 400X magnification using a BZ‐9000 microscope (Keyence, Osaka, Japan). The quantification of fluorescence intensity was performed using the Hybrid cell count BZ‐H2C software (Keyence). Based on the percentage of immunoreactive cells within the microscopic field of view, NeuGc Ag levels were classified into two groups: negative (0%) and positive (>0%) group. Four of the 66 initial hepatectomy samples were excluded because the quantification of the immunostaining Ag was impossible owing to poor storage conditions.

### 2.4 Reverse transcription‐quantitative polymerase chain reaction (RT‐qPCR)

The sections of the hepatectomized liver tissues were immediately stored in RNAlater® Tissue Protect Tubes (Qiagen, Hilden, Germany). After crushing the tissue with TissueLyser II (Qiagen), total RNA was extracted using the single‐step method of RNA isolation by acid guanidium thiocyanate‐phenol‐chloroform (Qiagen). Reverse transcription of total RNA was performed to generate the first‐strand cDNA. To determine the mRNA expression levels of human *CMAH* (*CMAHP*), the forward (5′ CCAGTCAGGAAGTC‐3′) (the lesion upper 92‐bp deletion) and reverse (5′‐GGTTGGAGGACCAG‐3′) (the lesion lower 92‐bp deletion) primers were designed according to the procedures for identifying the gene.^1^, ^3^ RT‐qPCR was performed with the following amplification profile: preincubation and initial activation at 94°C for 15 min, followed by 30 cycles of denaturation at 94°C for 1 min, primer annealing at 54°C for 1 min, and elongation at 72°C for 2 min. With β actin expression as reference, the average ∆Ct value of the whole group was determined. The relative quantification of mRNA expression was performed using the 2^‐∆∆^Ct method.^13^

### 2.5 Enzyme‐linked immunosorbent assay (ELISA)

The anti‐NeuGc Abs in the sera of patients with HCC and healthy volunteers were measured by ELISA, according to previously reported methods.^14^, ^15^ ELISA microplates (Costar 3591; Corning, New York, NY, USA) were coated with NeuGc‐ and NeuAc‐conjugated polyacrylamide (GlycoTech, Gaithersburg, MD, USA) overnight. We added peroxidase‐conjugated goat anti‐human IgG (Southern Biotech, Birmingham, AL, USA) or peroxidase‐conjugated mouse anti‐human IgG subclasses (Southern Biotech). The optical density was measured at 490 nm using the MTP 310 microplate reader (Corona Electric Co., Ltd., Ibaraki, Japan). We decided to analyze x100 and x500 dilution data based on the preliminary data (*n* = 16, Figure S1) in which the serum was diluted in 4 steps.

### 2.6 Statistical analysis

All statistical analyses were performed using the JMP® 14 software (SAS Institute Inc., Cary, NC, USA). The receiver operator characteristic (ROC) curve analysis was performed to determine the optimal cutoff values of anti‐NeuGc IgG Abs in the blood sera of the HCC patients and healthy volunteers and the area under the curve value was calculated. A multivariable logistic regression analysis was performed and the odds ratio (OR) and corresponding 95% confidence interval were calculated. Statistical analysis of the categorical data was performed using the Student's *t*‐test and Kruskal–Wallis test for continuous variables and the Pearson's chi‐squared test for categorical variables. Kaplan–Meier plots and log‐rank analysis were performed to determine the difference in overall survival (OS) and recurrent free survival (RFS) rate between the patient groups, with *P*‐values <.05 considered as statistically significant.

## 3 RESULTS

### 3.1 Identification of NeuGc Ag and CMAHP mRNA expressed on the HCC tissues

**FIGURE 1:**
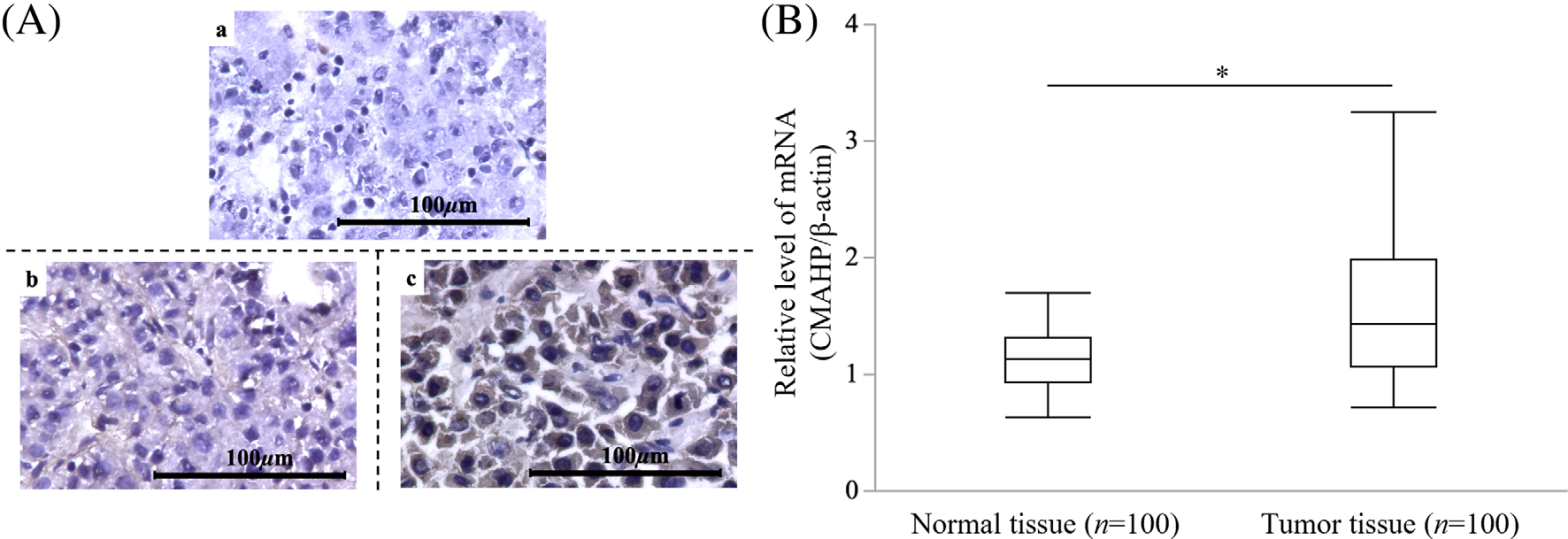
Identification of NeuGc Ag and *CMAHP* mRNA expressed on the HCC tissue. (A) Identification of *N*‐glycolylneuraminic acid (NeuGc) antigen (Ag) expressed on the hepatocellular carcinoma (HCC) tissues. These figures present the representative results for NeuGc Ag expression in the cell membrane: (a) Normal liver tissue (0%); (b) Negative tumor tissue (0%); (c) Positive tumor tissue (83%). (B) Identification of cytidine‐5′‐monophospho‐NeuAc hydroxylase pseudogene (*CMAHP*) mRNA expressed on the HCC tissues. The *CMAHP* mRNA expression was evaluated in the normal and tumor portions of the resected liver tissues from the hepatectomized HCC patients (*n* = 100). The tumor tissue had significantly higher *CMAHP* mRNA expression than the normal tissue (*P* < .01).

We evaluated NeuGc Ag expression in the resected HCC tissues from the patients who underwent hepatectomy for HCC by performing an immunochemistry assay on the normal and tumor regions of the tissues. Figure 1A presents the representative results for NeuGc Ag expression on the cell membrane: (a) NeuGc Ag‐negative normal liver tissue (0%); (b) NeuGc Ag‐negative tumor tissue (0%); (c) NeuGc Ag‐positive tumor tissue (83%). NeuGc Ag was not expressed in the normal regions of the liver tissue (*n* = 96, excluding the 4 cases). Regarding the tumor portion of the resected livers, 71 of the 96 HCC tissue samples (74.0%) expressed NeuGc Ag, whereas the other 25 did not.

*CMAHP*, which encodes a key enzyme in the synthesis of the sialic acids Neu5Ac and Neu5Gc in other mammals, was rendered a pseudogene in humans through an inactivating microdeletion and the subsequent fixation of the inactive allele in early human populations.^16^ The NeuGc expression in HCC tissues is likely due to the aberrant functionalization of *CMAHP*, leading to NeuGc biosynthesis. To address this possibility, we evaluated *CMAHP* mRNA expression normalized against β‐actin in the normal and tumor portions of the resected liver tissues from the hepatectomized HCC patients (*n* = 100). RT‐qPCR results revealed that the tumor tissues exhibited significantly higher *CMAHP* mRNA expression than the normal tissues (*P* < .01; Figure 1B).

### 3.2 Serum titers of anti‐NeuGc IgG Ab tended to be higher in the HCC patients than in the healthy volunteers

**FIGURE 2:**
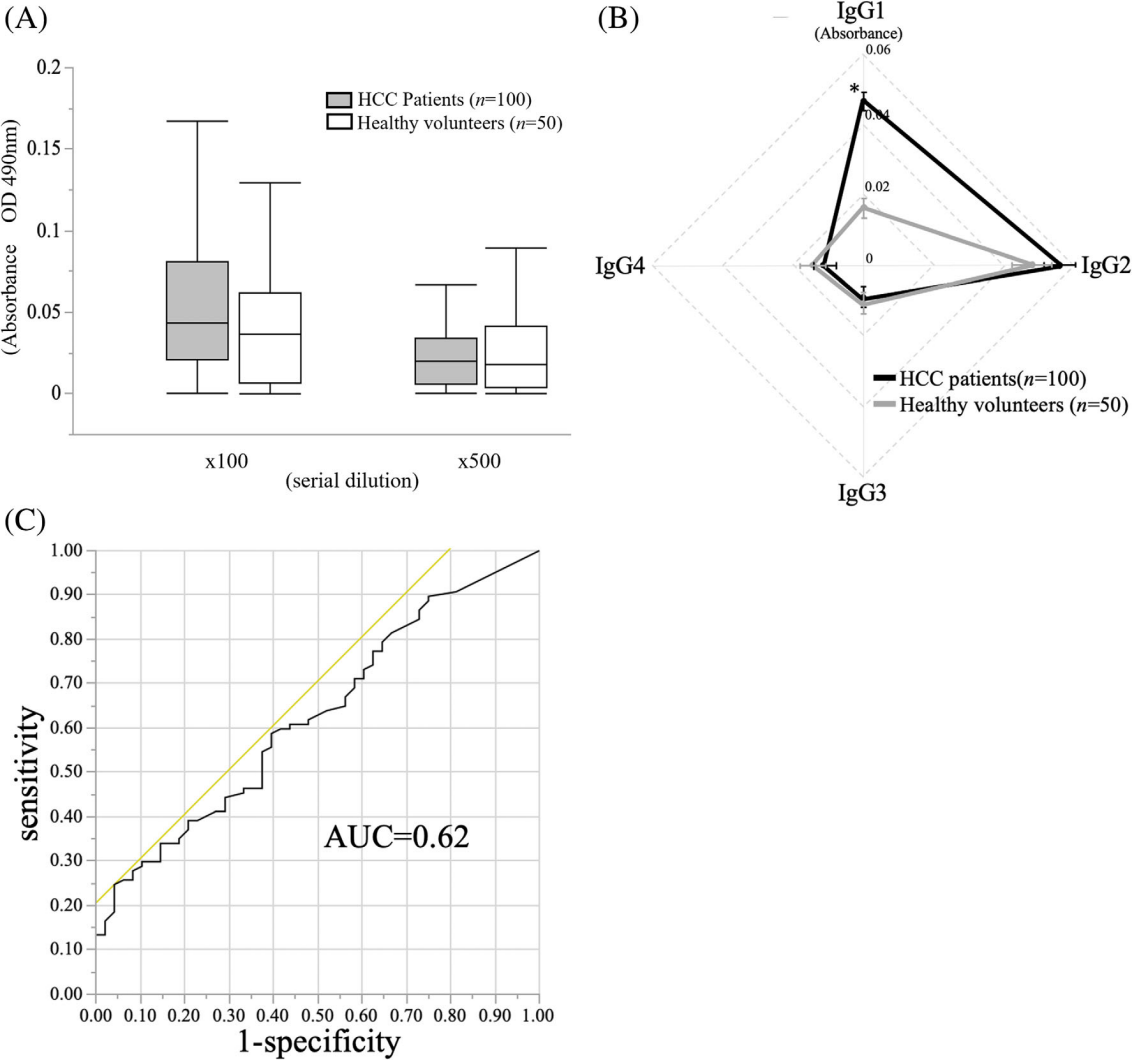
Serum titer of anti‐NeuGc IgG Ab in the HCC patients and healthy volunteers. (A) The average of anti‐NeuGc immunoglobulin (Ig) G antibody (Ab) titers tended to be higher in the sera of the HCC patients than in the healthy volunteers but no significant difference was observed between both groups (*P* = 0.089). (B) The titers of preoperative anti‐NeuGc IgG Ab subclasses in the HCC patients and healthy volunteers. The level of anti‐NeuGc IgG1 was significantly higher in the sera of the HCC patients than in the healthy volunteers (*P* < .01). (C) The optimal cutoff value of anti‐NeuGc IgG Ab between the HCC patients and healthy volunteers was calculated to be 0.04 by the receiver operator characteristic curve analysis.

The preoperative anti‐NeuGc IgG Ab titers in the sera of the HCC patients (*n* = 100) or healthy donors (*n* = 50) were measured by ELISA. The average of anti‐NeuGc IgG Ab titers tended to be higher in the sera of the HCC patients than in the healthy volunteers; however, the difference was not statistically significant (*P* = 0.089; Figure 2A). The titers of anti‐NeuGc IgG Ab subclasses were also measured to investigate the probable difference in the opsonic capacity of anti‐NeuGc IgG in the HCC patients and healthy volunteers. The results revealed that the level of anti‐NeuGc IgG1, which has high opsonizing activity (Ab‐dependent cellular cytotoxicity (ADCC) or complement‐dependent cytotoxicity (CDC)), was significantly higher in the sera of the HCC patients than in the healthy volunteers (*P* < .01; Figure 2B). By contrast, no significant differences were observed in other IgG subclasses. Using ROC analysis, the optimal cutoff value of anti‐NeuGc IgG Ab between the HCC patients and healthy volunteers was calculated to be 0.04. Thus, an absorbance value of 0.04 or higher was defined as anti‐NeuGc Ab positive (Figure 2C). Among healthy volunteers (*n* = 50), 19 had serum anti‐NeuGc IgG Abs above the cutoff value, resulting in a false positive rate of 38%. Since healthy individuals originally have NeuGc natural Abs, the false‐positive rate would have been relatively high.

### 3.3 Among patients hepatectomized for initial HCC, those with positive NeuGc Ag expression and the higher titers of preoperative anti‐NeuGc IgG Ab had a higher recurrence rate after the surgery

As the biological significance of NeuGc expression in tumor tissues and anti‐NeuGc IgG Ab titers in the sera may differ depending on the clinical course of HCC, we separately analyzed the potential of NeuGc as a biomarker in the patients with initial HCC (*n* = 66) and those with recurrent HCC (*n* = 34) (no overlap between the initial and recurrent patients). Among the patients who had undergone hepatectomy for initial HCC (the initial patient group) (*n* = 62, excluding the 4 cases), the classification of cases showing NeuGc Ag expression was as follows: negative, 18 cases and positive, 44 cases.

The clinical characteristics of the initial patient group are shown in Table 1. No significant differences were observed in the underlying diseases, tumor diameter, number of tumors, tumor marker levels of alpha‐fetoprotein (AFP), pathological vascular invasion, and the degree of differentiation between NeuGc Ag‐positive and ‐negative groups. However, protein induced by vitamin K absence‐II (PIVKAII) level, NeuGc Ab titer, and the recurrence rate within 2 years after hepatectomy in the NeuGc Ag‐positive group were significantly higher than those in the negative group (*P* < .05, <.01, and <.05, respectively).

**TABLE 1:** Clinical characteristics in patients who received hepatectomy for initial HCC (*n* = 62)

	NeuGc Ag expression level on HCC tissues		*P‐value*
	Positive (*n* = 44)	Negative (*n* = 18)	
Underlying disease (*n*)	HCV(27) HBV(7) Others(10)	HCV(9) HBV(2) Others(7)	N.S.
Tumor diameter, mm	36.6 ± 4.4	126.3 ± 99.1	N.S.
Numbers of tumor, *n*	2.0 ± 0.4	2.1 ± 0.5	N.S.
AFP, ng/mL	10220 ± 7190	47.5 ± 18.1	0.08
AFP L3, %	16.2 ± 3.4	9.9 ± 2.7	0.07
PIVKAII, mAU/mL	5350 ± 11909	548 ± 959	<0.05
Pathological vascular invasion			
Potal vein invasion [Vp (n)]	Vp3(2), Vp2(1), Vp1(8), Vp0(33)	Vp3(0), Vp2(0), Vp1(4), Vp0(14)	N.S
Hepatic vein invasion [Vv (n)]	Vv3(1), Vv2(2), Vv1(1), Vv0(40)	Vv3(0), Vv2(0), Vv1(2), Vv0(16)	N.S.
Hepatic artery invasion [Va (n)]	Va3(0), Va2(0), Va1(1), Va0(43)	Va3(0), Va2(0), Va1(0), Va0(18)	N.S.
Pathological stage (*n*)	IV(6) III(11) II(14)I(13)	IV(2) III(2) II(11)I(3)	N.S.
Differentiation (*n*)	well(9) moderately(29) poorly(6)	well(4) moderately(14) poorly(0)	N.S.
NeuGc titer (Absorbance)	0.06±0.01	0.03±0.01	<0.01
Numbers of recurrent cases within 2 years, *n* (%)	23 (52.3)	4 (22.2)	<0.05

*Note*: *P*‐values from Kruskal‐Wallis test for continuous data and Pearson's chi‐squared test for categorical data.

Abbreviations: *AFP*, alpha‐fetoprotein; *AFP L3*, lens culinaris agglutinin‐reactive fraction of alpha‐fetoprotein; *N.S*., Not significant; *PIVKAII*, protein induced by vitamin K absence‐II.

Next, within the initial patient group, we compared the clinical characteristics between the anti‐NeuGc IgG Ab‐positive (*n* = 35) and ‐negative patients (*n* = 31) (Table 2). The positive group exhibited a significantly higher lens culinaris agglutinin‐reactive fraction of AFP (AFP‐L3) level and recurrence rate within 2 years after hepatectomy than the negative group (*P* < .01 and <.001, respectively). Furthermore, the univariate analysis of risk factors revealed that progressed pathological stage, higher titers of anti‐NeuGc IgG Ab, and positive NeuGc Ag expression were significantly associated with the earlier recurrence of HCC (Table 3). Through multivariate analysis, the higher titer of anti‐NeuGc IgG Ab and positive NeuGc Ag expression were extracted as predictive factors with an OR of 4.9 and 6.3, respectively (Table 3).

**TABLE 2:** Clinical characteristics in patients who received hepatectomy for initial HCC (*n* = 66)

	Preoperative anti‐NeuGc IgG Ab		*P‐value*
	Positive (*n* = 35)	Negative (*n* = 31)	
Underlying disease (*n*)	HCV(20) HBV(4) Others(11)	HCV(20) HBV(6) Others(5)	N.S.
Tumor diameter, mm	29.0 ± 48	20.0 ± 3.9	N.S.
Numbers of tumor, *n*	2.4 ± 0.6	1.7 ± 0.3	N.S.
AFP, ng/mL	32.0 ± 8301	19.2 ± 4272	N.S.
AFP L3, %	9.7 ± 4.3	3.5 ± 1.8	<0.01
PIVKAII, mAU/mL	157.0 ± 1741	51.0 ± 1744	N.S.
Pathological vascular invasion			
Potal vein invasion [Vp (n)]	Vp3(1), Vp2(1), Vp1(7), Vp0(26)	Vp3(1), Vp2(0), Vp1(5), Vp0(25)	N.S^.^
Hepatic vein invasion [Vv (n)]	Vv3(0), Vv2(2), Vv1(2), Vv0(31)	Vv3(1), Vv2(0), Vv1(0), Vv0(30)	N.S.
Hepatic artery invasion [Va (n)]	Va3(0), Va2(0), Va1(1), Va0(34)	Va3(0), Va2(0), Va1(0), Va0(31)	N.S.
Pathological stage (*n*)	IV(6) III(10) II(10)I(9)	IV(2) III(5) II(16)I(8)	N.S.
Differentiation (*n*)	well(7) moderately(23) poorly(5)	well(6) moderately(23) poorly(2)	N.S.
Numbers of recurrent cases within 2 years, *n* (%)	22 (62.9)	6 (19.4)	<0.001

*Note*: *P*‐values from Kruskal‐Wallis test for continuous data and Pearson's chi‐squared test for categorical data.

Abbreviations: *AFP*, alpha‐fetoprotein; *AFP L3*, lens culinaris agglutinin‐reactive fraction of alpha‐fetoprotein; *N.S*., Not significant; *PIVKAII*, protein induced by vitamin K absence‐II.

**TABLE 3:** Predictive factors relating to earlier recurrence within 2 years in hepatectomized patients for initial HCC (*n* = 66)

	Univariate analysis	Multivariate analysis	Odds ratio (95% CI)
Tumor diameter	0.43	‐	‐
Numbers of tumor	0.49	‐	‐
AFP	0.71	‐	‐
AFP L3	0.58	‐	‐
PIVKAII	0.17	‐	‐
Pathological vascular invasion			
Potal vein invasion [Vp]	0.61	‐	‐
Hepatic vein invasion [Vv]	0.26	‐	‐
Hepatic artery invasion [Va]	0.18	‐	‐
Pathological stage	0.02	0.78	1.3 (0.2‐7.5)
Differentiation	0.13	‐	‐
NeuGc titers (Absorbance)	0.0002	0.03	4.9 (1.2‐21.0)
Positive NeuGc Ag expression	<0.0001	0.007	6.3 (0.5‐77.0)

*Note*: *P*‐values from Kruskal‐Wallis test for continuous data and Pearson's chi‐squared test for categorical data.

Abbreviations: *AFP*, alpha‐fetoprotein; *AFP L3*, lens culinaris agglutinin‐reactive fraction of alpha‐fetoprotein; *N.S*., Not significant; *PIVKAII*, protein induced by vitamin K absence‐II.

### 3.4 NeuGc expression in the HCC tissues was closely related to serum anti‐NeuGc IgG titers in the initial patient group

**FIGURE 3:**
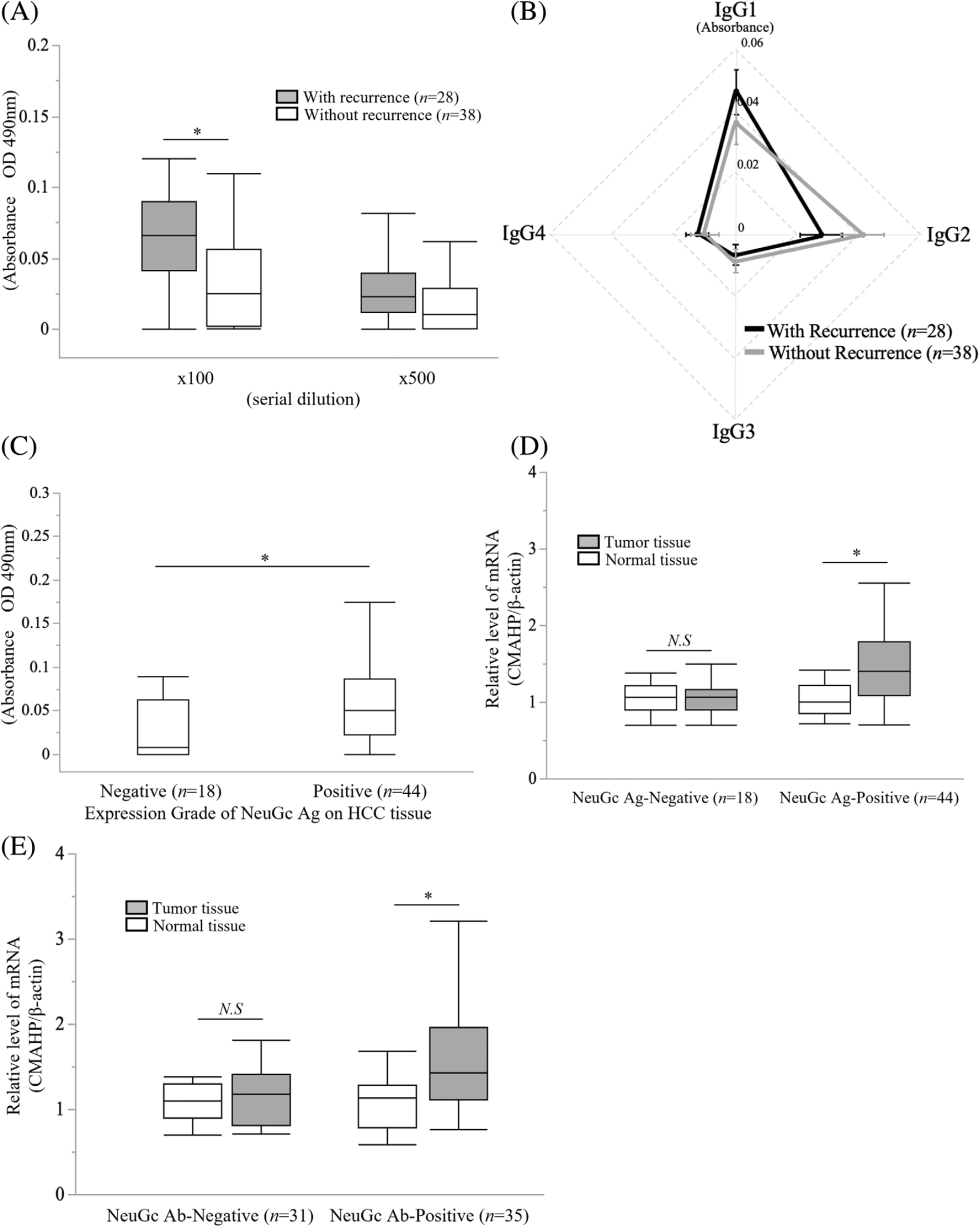
Association of NeuGc expression in HCC tissues with serum anti‐NeuGc IgG titers in initial patient. (A) The titers of anti‐*N*‐glycolylneuraminic acid (NeuGc) immunoglobulin (Ig) G antibody (Ab) in the patients with (*n* = 28) and without (*n* = 38) hepatocellular carcinoma (HCC) recurrence within 2 years after hepatectomy for initial HCC. The titers of anti‐NeuGc IgG Ab were significantly higher in the patients with recurrence than in those without recurrence (*P* < .01). (B) The titers of anti‐NeuGc IgG Ab subclasses in the patients with and without HCC recurrence in the initial HCC patient group. No significant difference was observed between the patients with (*n* = 28) and without (*n* = 38) HCC recurrence within 2 years after hepatectomy for initial HCC. (C) The relationship between NeuGc antigen (Ag) expression in the HCC tissues and anti‐NeuGc Ab titers in the initial HCC patient group. The positive NeuGc Ag group (>0%; *n* = 44) had significantly higher IgG Ab titers than the negative group (0%; *n* = 18, *P* < .01). (D) The relationship between the relative cytidine‐5′‐monophospho‐NeuAc hydroxylase pseudogene (*CMAHP*) mRNA expression and NeuGc Ag expression in the initial HCC patient group. The *CMAHP* mRNA expression was significantly higher in the tumor tissues of the positive NeuGc Ag group than in the normal tissues (*P* < .05). (E) The relationship between the relative *CMAHP* mRNA expression and anti‐NeuGc IgG Ab in the initial HCC patient group. The tumor tissue had significantly higher levels of *CMAHP* mRNA than the normal tissues in the preoperative anti‐NeuGc Ab‐positive group *(P* < .01).

We compared the titers of preoperative anti NeuGc IgG Ab between the patients with (*n* = 28) and without (*n* = 38) HCC recurrence within 2 years after hepatectomy for initial HCC. The titers of anti‐NeuGc Ab were significantly higher in the patients with recurrence than in those without recurrence (*P* < .01; Figure 3A). We also compared the titers of anti NeuGc IgG Ab subclasses and found no significant difference between the patients with and without HCC recurrence (Figure 3B). Next, we investigated the relationship between NeuGc Ag expression in the HCC tissues and anti‐NeuGc Ab titers. The results revealed that the NeuGc Ag‐positive group (*n* = 44) had significantly higher anti‐NeuGc Ab titers than the negative group (*n* = 18, *P* < .01) (Figure 3C). This result suggested that the increased titers of preoperative anti‐NeuGc IgG Ab reflected whether the NeuGc Ag was expressed in HCC tissues.

*CMAHP* mRNA expression was significantly higher in the tumor tissues of the NeuGc Ag‐positive group than in the normal tissues (*P* < .05; Figure 3D). Consistently, the tumor tissues had significantly higher levels of *CMAHP* mRNA than the normal tissues in the anti‐NeuGc Ab‐positive group (*P* < .01; Figure 3E), whereas no significant difference was observed between the normal and tumor tissues in the NeuGc Ag and Ab‐negative group.

### 3.5 Among patients hepatectomized for recurrent HCC, those with positive NeuGc Ag expression and higher preoperative anti‐NeuGc IgG Ab titers had a higher recurrence rate after the surgery

As NeuGc Ag and Ab were shown to be involved in the earlier recurrence of HCC, we investigated whether NeuGc Ag or Ab is a factor that contributes to repeated recurrence only in cases that were hepatectomized for recurrent HCC. In the group of patients who had undergone hepatectomy for recurrent HCC (the recurrent patient group) (*n* = 34), the classification of cases showing NeuGc Ag expression was as follows: negative, 7 cases, and positive, 27 cases. The clinical characteristics of the recurrent patient group are shown in Table 4, 5, 6. PIVKAII level and the recurrence rate within 2 years after hepatectomy were significantly higher in the NeuGc Ag‐positive group than in the negative group (*P* < .05) (Table 4). The anti‐NeuGc Ab positive group exhibited a significantly higher AFP‐L3 level and earlier recurrence rate after hepatectomy (*P* < .05) (Table 5). No significant differences were observed for other characteristics. Both univariate and multivariate analyses of predictive factors revealed that the positive NeuGc Ag expression was significantly associated with the previous recurrence of HCC in the recurrent patient group with an OR of 14.0 (Table 6).

**TABLE 4:** Clinical characteristics in patients who received hepatectomy for recurrent HCC (*n* = 34)

	NeuGc Ag expression level on HCC tissues		*P‐value*
	Positive (*n* = 27)	Negative (*n* = 7)	
Underlying disease (*n*)	HCV(17) HBV(8) Others(2)	HCV(4) HBV(3) Others(0)	N.S.
Tumor diameter, mm	19.0 ± 2.8	19.1 ± 2.0	N.S.
Numbers of tumor, *n*	1.4 ± 0.1	1.3 ± 0.2	N.S.
AFP, ng/mL	46.1 ± 23.3	112.7 ± 94.9	N.S.
AFP L3, %	8.2 ± 3.0	3.4 ± 1.3	0.08
PIVKAII, mAU/mL	110.7 ± 44.8	21.6 ± 5.5	<0.05
Pathological vascular invasion			
Potal vein invasion [Vp (n)]	Vp3(1), Vp2(0), Vp1(0), Vp0(26)	Vp3(0), Vp2(0), Vp1(1), Vp0(6)	N.S
Hepatic vein invasion [Vv (n)]	Vv3(0), Vv2(0), Vv1(0), Vv0(27)	Vv3(0), Vv2(0), Vv1(0), Vv0(7)	N.S.
Hepatic artery invasion [Va (n)]	Va3(0), Va2(0), Va1(0), Va0(27)	Va3(0), Va2(0), Va1(0), Va0(7)	N.S.
Pathological stage (*n*)	IV(0) III(6) II(10)I(11)	IV(0) III(3) II(2)I(2)	N.S.
Differentiation (*n*)	well(3) moderately(23) poorly(1)	well(1) moderately(6) poorly(0)	N.S.
NeuGc titer (Absorbance)	0.09 ± 0.02	0.06 ± 0.02	N.S.
Numbers of recurrent cases within 2 years, *n* (%)	19 (70.3)	2 (28.6)	<0.05

*Note*: *P*‐values from Kruskal‐Wallis test for continuous data and Pearson's chi‐squared test for categorical data.

Abbreviations: *AFP*, alpha‐fetoprotein; *AFP L3*, lens culinaris agglutinin‐reactive fraction of alpha‐fetoprotein; *N.S*., Not significant; *PIVKAII*, protein induced by vitamin K absence‐II.

**TABLE 5:** Clinical Characteristics in patients who received hepatectomy for recurrent HCC (*n* = 34)

	Preoperative anti‐NeuGc IgG Ab		*P‐value*
	Positive (*n* = 22)	Negative (*n* = 12)	
Underlying disease (*n*)	HCV(12) HBV(8) Others(3)	HCV(8) HBV(4) Others(0)	N.S.
Tumor diameter, mm	17.0 ± 3.3	16.0 ± 1.8	N.S.
Numbers of tumor, *n*	1.5 ± 0.1	1.25 ± 0.1	N.S.
AFP, ng/mL	7.1 ± 39.8	5.2 ± 14.7	N.S.
AFP L3, %	5.0 ± 3.5	0.5 ± 0.5	<0.05
PIVKAII, mAU/mL	18.5 ± 54.8	19.0 ± 12.7	N.S.
Pathological vascular invasion			
Potal vein invasion [Vp (n)]	Vp3(1), Vp2(0), Vp1(0), Vp0(21)	Vp3(0), Vp2(0), Vp1(1), Vp0(11)	N.S^.^
Hepatic vein invasion [Vv (n)]	Vv3(0), Vv2(0), Vv1(0), Vv0(22)	Vv3(0), Vv2(0), Vv1(0), Vv0(12)	N.S.
Hepatic artery invasion [Va (n)]	Va3(0), Va2(0), Va1(0), Va0(22)	Va3(0), Va2(0), Va1(0), Va0(12)	N.S.
Pathological stage (*n*)	IV(0) III(7) II(9) I(7)	IV(0) III(2) II(3)I(7)	N.S.
Differentiation (*n*)	well(3) moderately(19) poorly(0)	well(1) moderately(10) poorly(1)	N.S.
Numbers of recurrent cases within 2 years, *n* (%)	17 (77.3)	4 (33.3)	<0.05

*Note*: *P*‐values from Kruskal‐Wallis test for continuous data and Pearson's chi‐squared test for categorical data.

Abbreviations: *AFP*, alpha‐fetoprotein; *AFP L3*, lens culinaris agglutinin‐reactive fraction of alpha‐fetoprotein; *N.S*., Not significant; *PIVKAII*, protein induced by vitamin K absence‐II.

**TABLE 6:** Predictive factors relating to earlier recurrence within 2 years in hepatectomized patients for recurrent HCC (*n* = 34)

	Univariate analysis	Multivariate analysis	Odds ratio (95% CI)
Tumor diameter	0.29	‐	‐
Numbers of tumor	0.81	‐	‐
AFP	0.51	‐	‐
AFP L3	0.02	0.17	0.9 (0.8‐1.1)
PIVKAII	0.1	‐	‐
Pathological vascular invasion			
Potal vein invasion [Vp]	0.23	‐	‐
Hepatic vein invasion [Vv]	N/A	‐	‐
Hepatic artery invasion [Va]	N/A	‐	‐
Pathological stage	0.87	‐	‐
Differentiation	0.28	‐	‐
NeuGc titers (Absorbance)	0.01	0.09	3.8 (0.4‐33.6)
Positive NeuGc Ag expression	<0.001	0.01	14.0 (1.4‐136)

*Note*: *P*‐values from Kruskal‐Wallis test for continuous data and Pearson's chi‐squared test for categorical data.

Abbreviations: *AFP*, alpha‐fetoprotein; *AFP L3*, lens culinaris agglutinin‐reactive fraction of alpha‐fetoprotein; *N.S*., Not significant; *PIVKAII*, protein induced by vitamin K absence‐II.

**FIGURE 4:**
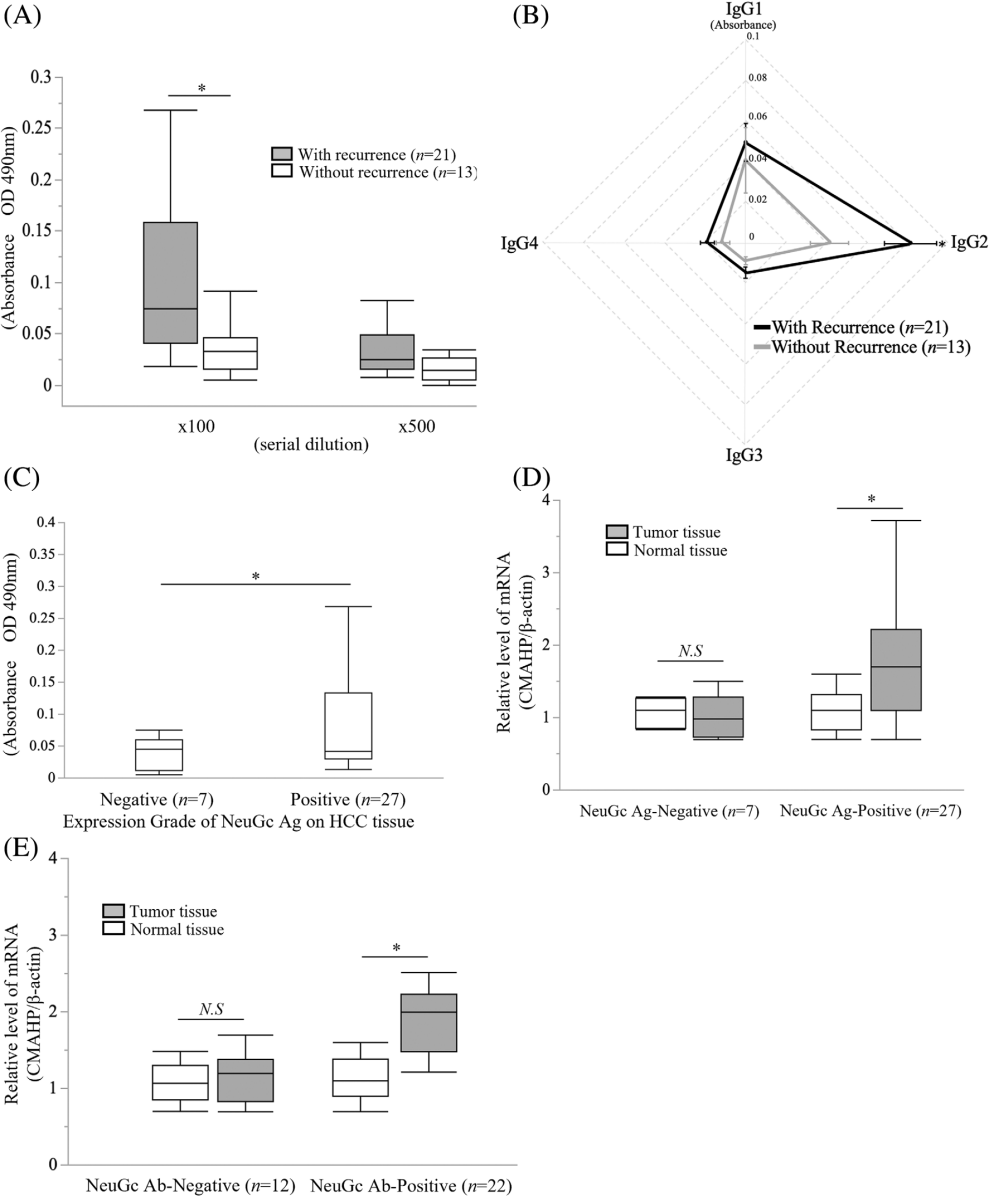
Association of NeuGc expression in HCC tissues with serum anti‐NeuGc IgG titers in recurrent patient. (A) The titers of anti‐*N*‐glycolylneuraminic acid (NeuGc) immunoglobulin (Ig) G antibody (Ab) in the patients with (*n* = 21) and without (*n* = 13) hepatocellular carcinoma (HCC) recurrence within 2 years after hepatectomy for recurrent HCC. The anti‐NeuGc IgG Ab titers were significantly higher in the patients with HCC recurrence (*P* < .01). (B) The titers of anti NeuGc IgG Ab subclasses in the patients with and without HCC recurrence in the recurrent HCC patient group. The IgG2 titer was significantly higher in the patients with early recurrence than in the patients without early recurrence (*P* < .05). No significant differences were observed in other IgG subclasses. (C) The relationship between NeuGc antigen (Ag) expression in the HCC tissues and anti‐NeuGc Ab titers in the recurrent HCC patient group. The positive NeuGc Ag group had a significantly higher IgG titer than the negative group (*P* < .05). (D) The relationship between the relative *CMAHP* mRNA expression and NeuGc Ag expression in the recurrent HCC patient group. The positive NeuGc Ag group showed significantly higher *CMAHP* mRNA expression in the tumor tissues than that in the normal tissues (*P* < .05). (E) The relationship between the relative cytidine‐5′‐monophospho‐NeuAc hydroxylase pseudogene (*CMAHP*) mRNA expression and anti‐NeuGc IgG Ab in the recurrent HCC patient group. The anti‐NeuGc Ab positive group showed significantly higher *CMAHP* mRNA expression in the tumor tissues than in the normal tissues (*P* < .05).

Comparison of the preoperative titers of anti‐NeuGc IgG Ab in the patients with (*n* = 21) and without (*n* = 13) HCC recurrence within 2 years after hepatectomy revealed that the anti‐NeuGc IgG Ab titers were significantly higher in the patients with HCC recurrence (*P* < .01; Figure 4A). We further compared the titers of anti NeuGc IgG Ab subclasses between the patients with and without HCC recurrence (Figure 4B). The IgG2 titer, which has a low opsonizing capacity, was significantly higher in the patients with early recurrence than in the patients without early recurrence (*P* < .05). No significant differences were observed in other IgG subclasses. Thus, patients with earlier recurrence after hepatectomy for recurrent HCC might possess immune systems incapable of spontaneously eliminating NeuGc‐expressing tumor cells.

We further investigated NeuGc Ag expression in the hepatectomized tumor tissues in the recurrent HCC patient group. Similar to the results for the initial HCC patient group, the NeuGc Ag‐positive group had significantly increased titers of anti‐NeuGc IgG Ab (*P* < .05; Figure 4C).

We compared the relative *CMAHP* mRNA expression in the recurrent HCC patient group. The NeuGc Ag‐positive group exhibited a significantly higher *CMAHP* mRNA expression in the tumor tissues than that in the normal tissues (*P* < .05) (Figure 4D). The *CMAHP* mRNA expression in the anti‐NeuGc Ab‐positive group was also significantly higher in the tumor tissues than in the normal tissues (Figure 4E).

### 3.6 Positive NeuGc Ag expression and the higher titers of preoperative anti‐NeuGc Ab were associated with significantly reduced RFS of the patients with initial hepatectomy

**FIGURE 5:**
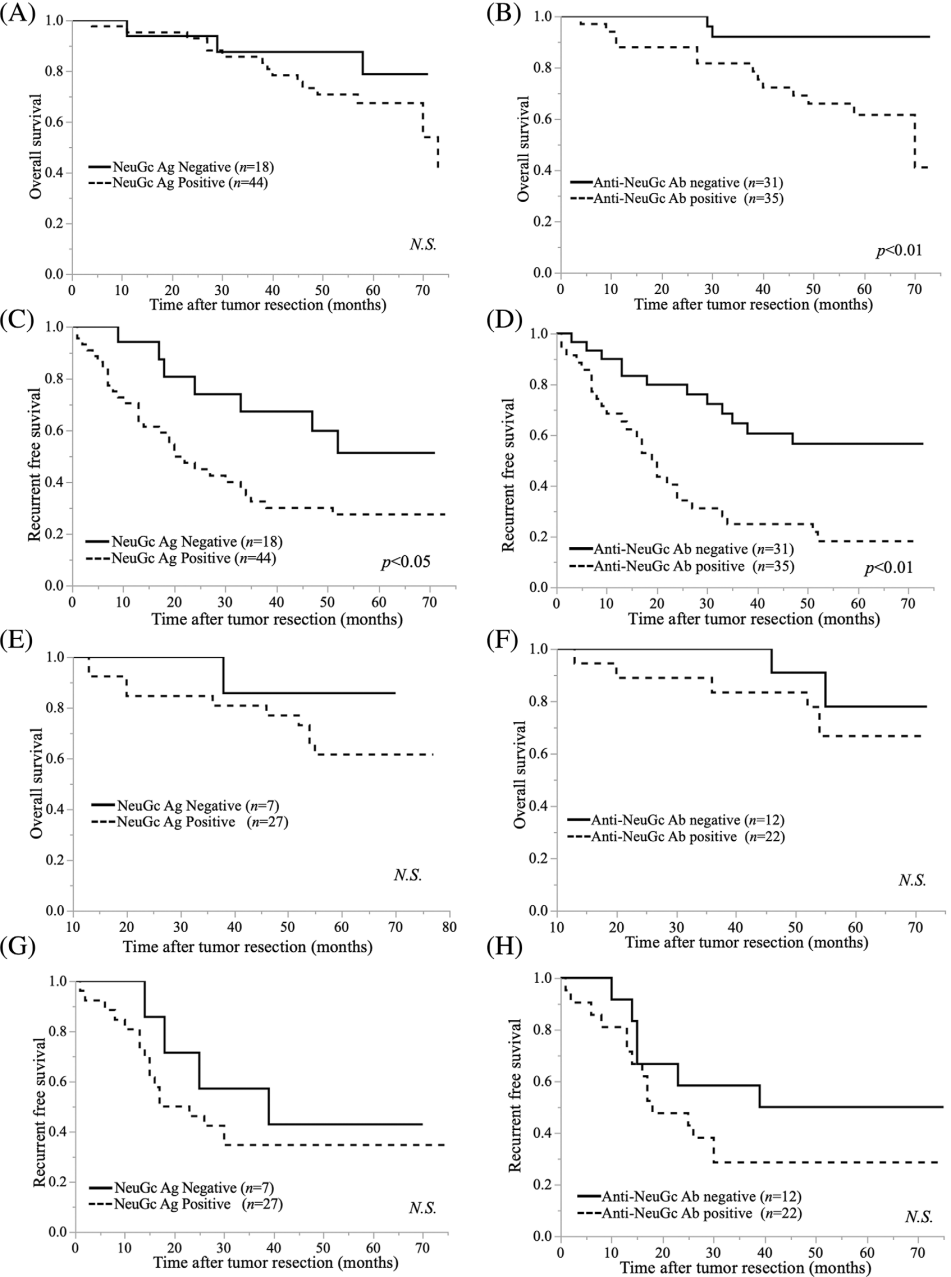
Association between positive NeuGc Ag and Anti‐NeuGc Ab OS and RFS rate with hepatectomy. (A) The overall survival (OS) rate in the initial hepatocellular carcinoma (HCC) patient group, in both the positive and negative *N*‐glycolylneuraminic acid (NeuGc) antigen (Ag) expression groups. The positive group exhibited shorter OS than the negative group without any significant difference. (B) The OS rate in the initial HCC patient group between the anti‐NeuGc antibody (Ab)‐positive and ‐negative groups. The anti‐NeuGc Ab‐positive subgroup showed significantly reduced OS compared with the anti‐NeuGc Ab‐negative group (*P* < .01). (C) The recurrent free survival (RFS) rate in the initial HCC patient group, in both the positive and negative NeuGc Ag expression groups. The positive group exhibited significantly shorter OS than the negative group (*P* < .05). (D) The RFS rate in the initial HCC patient group between anti‐NeuGc Ab‐positive and ‐negative groups. The anti‐NeuGc Ab‐positive group showed significantly reduced OS compared with the anti‐NeuGc Ab‐negative group (*P* < .01). (E‐H) The OS (Figure 5E) and RFS (Figure 5G) rates in the recurrent HCC patient group, in both the positive and negative NeuGc Ag expression groups. The positive group exhibited shorter OS than the negative group without any significant difference. The OS (Figure 5F) and RFS (Figure 5H) rate in the recurrent HCC patient group between anti‐NeuGc Ab‐positive and ‐negative groups. No significant difference was observed between both groups.

We compared the OS rate in the initial patient group between the NeuGc Ag‐positive and ‐negative groups. The positive group exhibited shorter OS (not significant, Figure 5A) and RFS (*P* < .05; Figure 5C) than the negative group. Next, we observed that the anti‐NeuGc Ab‐positive group exhibited a significantly reduced OS (*P* < .01; Figure 5B) and RFS (*P* < .01; Figure 5D) compared with the negative group.

We also compared the OS of the recurrent patient group between the NeuGc Ag‐positive and ‐negative groups. The results showed that the OS and RFS rate did not show a significant difference. However, the positive group tended to have shorter OS and RFS than the negative group (Figure 5E and G). The OS and RFS of patients hepatectomized for recurrent HCC were not significantly different between the anti‐NeuGc IgG Ab‐positive and ‐negative groups, whereas the positive group tended to exhibit a reduced OS and RFS rate (Figure 5F and H).

## 4 DISCUSSION

Despite optimal treatment, HCC has a high recurrence rate even after curative surgery. It recurs in 50%–80% of patients following resection, with a majority of recurrences developing within 2 years.^17^ Previous studies have shown that the early recurrence of HCC after resection, which is associated with increased tumor size, number of tumors, and portal vein invasion, results in low OS.^18^, ^19^, ^20^, ^21^ Several studies have reported that serological biomarkers are useful in predicting HCC recurrence risk. However, the serum biomarkers for HCC exhibit inadequate sensitivity for predicting recurrence.^22^

Sialic acids play fundamental roles in cell–cell and cell–microenvironment interactions such as immune responses.^23^ The most common sialic acids in mammals, NeuAc and NeuGc, have a structural difference of a single oxygen atom at the C5 position.^24^ In general, normal human cells do not express NeuGc because of the inactivation of functional *CMAH* caused by a mutation in *CMAH*.^25^ Higashi et al. and Merrick et al. have reported the characterization of Abs against Hanganutziu–Deicher (H–D) Ag as gangliosides containing NeuGc.^26^, ^27^ H–D Abs containing NeuGc have been discovered in the blood sera of lung, colon, breast cancer, melanoma, and HCC patients.^6^, ^7^ Koda et al. reported that increased serum levels of anti‐NeuGc Abs were observed in patients with HCC and the presence of anti‐NeuGc Abs was mainly attributed to the expression of NeuGc in HCC cells.^7^

In the present study, we identified the expression of NeuGc Ag in most HCC tissues and the expression of *CMAHP* mRNA in the NeuGc Ag‐positive tissues. *CMAHP* amplification in NeuGc Ag‐expressing HCC tissues likely indicates that either *CMAHP* is transformed into a functional *CMAH* that biosynthesizes NeuGc or *CMAHP* is mutated; however, such hypotheses remain speculative. Samraj et al. reported the mechanism of incorporating non‐human sialic acid NeuGc into human tissues and its potential effect on cancer initiation and progression and concluded that the reduced consumption of NeuGc‐containing food may have a major effect in preventing the development of malignant diseases.^28^ Further studies on the biology of *CMAHP* and NeuGc Ag‐expression in HCC tissues and circulating anti‐NeuGc Abs are required to completely elucidate the mechanism of HCC carcinogenesis.

Regardless of the mechanism, the intra‐tumoral expression of NeuGc Ag was associated with postoperative recurrence and prognosis in the HCC patient groups. Hence, examining NeuGc Ag expression and relative *CMAHP* mRNA levels in HCC tissues may be useful in identifying high‐risk groups for HCC recurrence and prognosis. However, as liver tissues are only available after surgery, no way is available to determine the risk of postoperative recurrence before surgery. As a factor that can predict the risk of postoperative recurrence preoperatively, which is valuable information for determining surgical indications, we investigated the effect of NeuGc Ab titers in the preoperative sera of the HCC patients, which were well correlated with NeuGc Ag expression or *CMAHP* mRNA level, on prognosis. The analysis of oncological characteristics showed a significant difference between the anti‐NeuGc Ab‐positive and ‐negative groups of the HCC patients who underwent hepatectomy with early recurrence within 2 years, and no significant correlation was found between HBV or HCV infection and the results of NeuGc antigen expression, NeuGc antibody titers, and *CMAHP* mRNA level. (Table 2 and 5). These results indicated that the serum anti‐NeuGc Ab can be used as a risk factor for predicting HCC recurrence at an early stage after hepatectomy; however, its potential benefit as a tumor marker monitored regularly after surgery for speculating HCC recurrence remains entirely unknown. We additionally found that NeuGc Ag expression in the HCC tissues and NeuGc Ab titers in the perioperative sera of the HCC patients were correlated (Figure 3C and 4C). The higher anti‐NeuGc Ab titer in the sera of patients with HCC and positive NeuGc Ag expression appears to indicate that humoral immunity, a biological defense mechanism, targets cancer antigens, leading to the prevention of HCC recurrence. Consistent with the speculative conviction, we showed that patients with anti‐NeuGc Ab in their sera showed a high HCC recurrence rate at an early stage after hepatectomy and poor prognosis. Thus, NeuGc Ag expression in the HCC tissues and the presence of anti‐NeuGc Ab in the HCC patients were associated with promoting rather than suppressing HCC recurrence; however, the reason for this contradiction could not be clarified in this study.

Anti‐NeuGc IgG subclasses were evaluated to see if the NeuGc Ab was of a type that could eliminate cancer cells. Human IgG is classified into four subclasses.^29^ Both IgG1 and IgG3 have high‐affinity binding to most fragment‐crystallizable region gamma receptors (FcγRs); they are critical activators of ADCC, CDC, and Ab‐dependent cell phagocytosis.^30^ IgG4 only has a high affinity to FcγRs, and it is a poor inducer of Fc‐mediated effector functions, whereas IgG2 has a weak affinity to all FcγRs.^29^ As shown in Figure 2B, the healthy volunteers exhibited anti‐NeuGc IgG predominantly in IgG2, whereas the HCC patients exhibited a significantly increased IgG1 level. In the patients with recurrent HCC, the IgG2 subclass was significantly promoted in subsequent recurrent cases than in the cases without another recurrence (Figure 4B). Thus, anti‐NeuGc Abs switch to a subclass with weak opsonizing effects on HCC recurrence is. Further studies are needed to clarify how HCC recurrence is associated with class switching of Abs that respond to cancer glycan antigens.

In this study, we investigated the relationship between the NeuGc Ag on the resected specimen and the NeuGc Ab titer at the time of resection. Therefore, measuring the serum NeuGc Ab may predict early recurrence and prognosis even in non‐hepatic resection cases. On the other hand, since we have not investigated temporal kinetics in NeuGc Ab titers after hepatic resection, it may not be a biomarker in the true sense.

## 5 CONCLUSIONS

Our findings suggested that preoperative anti‐NeuGc Ab titers and positive NeuGc Ag expression in resected HCC tissues can predict the early recurrence and prognosis of HCC after curative hepatectomy. Therefore, these factors may serve as potential predictive factors for future studies on improving HCC prognosis and therapeutics.

## AUTHOR CONTRIBUTIONS

**Shuji Akimoto:** Data curation (lead); formal analysis (lead); investigation (lead); methodology (lead); writing – original draft (lead); writing – review and editing (equal). **Hiroyuki Tahara:** Conceptualization (lead); funding acquisition (equal); investigation (equal); project administration (lead); supervision (equal); writing – original draft (equal); writing – review and editing (equal). **Senichiro Yanagawa:** Data curation (supporting); formal analysis (supporting); investigation (supporting). **Kentaro Ide:** Data curation (supporting); project administration (supporting); supervision (supporting). **Yuka Tanaka:** Data curation (supporting); project administration (supporting); supervision (supporting). **Tsuyoshi Kobayashi:** Data curation (supporting). **Hideki Ohdan:** Conceptualization (equal); funding acquisition (equal); project administration (equal); supervision (equal); validation (equal); writing – review and editing (supporting).

## FUNDING INFORMATION

This study was funded by the Medical Research Grants, a grant from the Takeda Science Foundation 2018. This work was supported in part by the JSPS KAKENHI Grant number JP19H01057 and AMED under Grant Numbers JP20fk0210051.

## CONFLICT OF INTEREST STATEMENT

The authors declare that they have no competing interests.

## ETHICS STATEMENT

The protocol for this research project has been approved by a suitably constituted Ethics Committee of the institution and it conforms to the provisions of the Declaration of Helsinki. Committee of Hiroshima University Hospital, Approval No. E‐1580.

## Supporting information


**Figure S1.** The titers of preoperative anti‐NeuGc IgG Ab with four step serial dilution in the HCC patients and healthy volunteers ‐preliminary data‐

## Data Availability

Data sharing is not applicable to this article as no new data were created or analyzed in this study.
