# Supplementary material for: Heterophile carbohydrate antigen N‐glycolylneuraminic acid as a potential biomarker in patients with hepatocellular carcinoma
Source: Cancer Rep (Hoboken). 2023 Jun 2;6(8):e1831. doi: 10.1002/cnr2.1831 (PMC10432449; doi:10.1002/cnr2.1831)

**Fig.S1 The titers of preoperative anti-NeuGc IgG Ab with four step serial dilution in the HCC patients and healthy volunteers (preliminary data)**


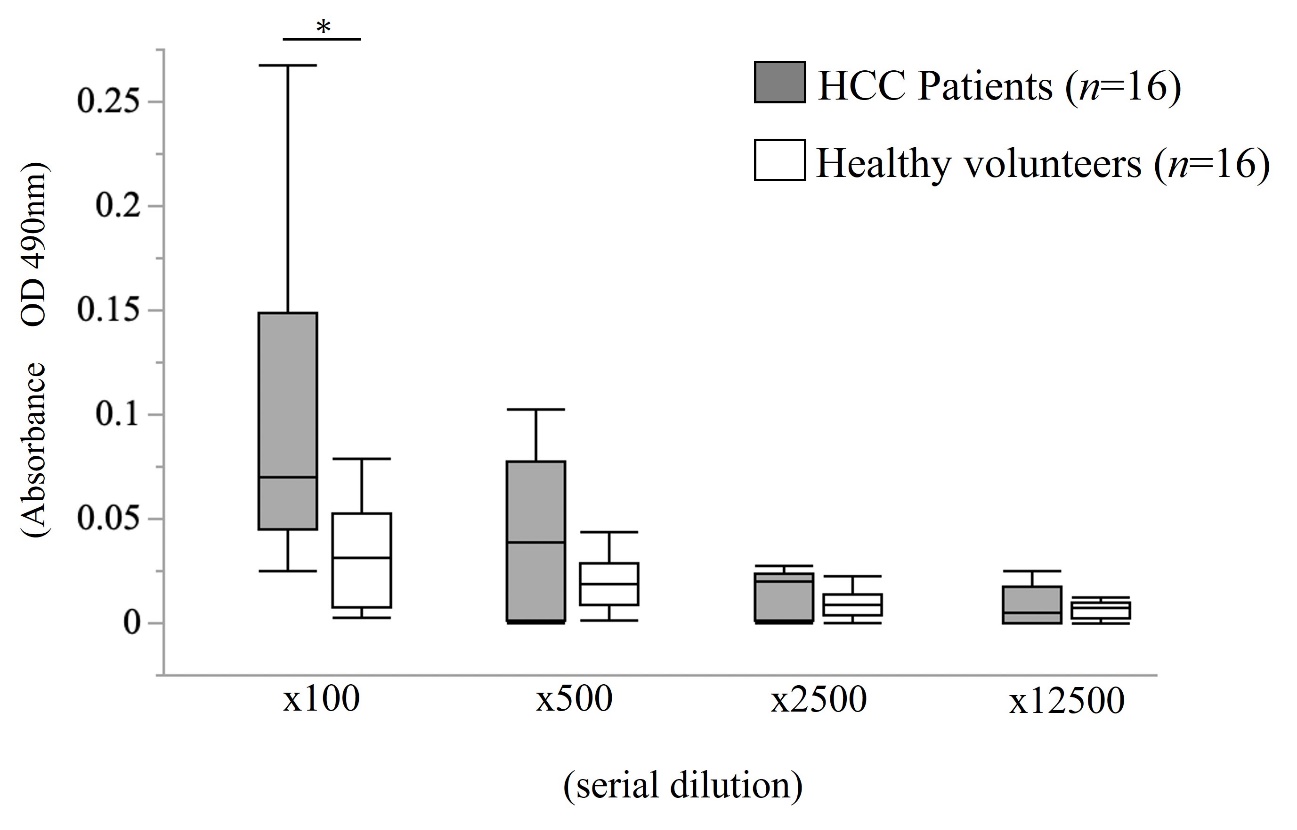

Supplement: Supplementary file 1 — Figure S1. The titers of preoperative anti‐NeuGc IgG Ab with four step serial dilution in the HCC patients and healthy volunteers ‐preliminary data‐ [file CNR2-6-e1831-s001.docx]
